# Ultrafast DNA Amplification Using Microchannel Flow-Through PCR Device

**DOI:** 10.3390/bios12050303

**Published:** 2022-05-06

**Authors:** Yen-Heng Lin, Xiang-Jun Liao, Wei Chang, Chiuan-Chian Chiou

**Affiliations:** 1 Graduate Institute of Biomedical Engineering, Chang Gung University, Taoyuan 333, Taiwan; hanerbill@gmail.com; 2 Department of Laboratory Medicine, Chang Gung Memorial Hospital, Taoyuan 333, Taiwan; 3 Master and PhD Program in Biotechnology Industry, College of Medicine, Chang Gung University, Taoyuan 333, Taiwan; deamit114@gmail.com; 4 Department of Medical Biotechnology and Laboratory Science, College of Medicine, Chang Gung University, Taoyuan 333, Taiwan; 5 Department of Thoracic Medicine, Chang Gung Memorial Hospital, Taoyuan 333, Taiwan

**Keywords:** ultrafast polymerase chain reaction, microchannel, flow-through PCR, Klen Taq

## Abstract

Polymerase chain reaction (PCR) is limited by the long reaction time for point-of-care. Currently, commercial benchtop rapid PCR requires 30–40 min, and this time is limited by the absence of rapid and stable heating and cooling platforms rather than the biochemical reaction kinetics. This study develops an ultrafast PCR (<3 min) platform using flow-through microchannel chips. An actin gene amplicon with a length of 151 base-pairs in the whole genome was used to verify the ultrafast PCR microfluidic chip. The results demonstrated that the channel of 56 μm height can provide fast heat conduction and the channel length should not be short. Under certain denaturation and annealing/extension times, a short channel design will cause the sample to drive slowly in the microchannel with insufficient pressure in the channel, causing the fluid to generate bubbles in the high-temperature zone and subsequently destabilizing the flow. The chips used in the experiment can complete 40 thermal cycles within 160 s through a design with the 56 µm channel height and with each thermal circle measuring 4 cm long. The calculation shows that the DNA extension speed is ~60 base-pairs/s, which is consistent with the theoretical speed of the Klen Taq extension used, and the detection limit can reach 67 copies. The heat transfer time of the reagent on this platform is very short. The simple chip design and fabrication are suitable for the development of commercial ultrafast PCR chips.

## 1. Introduction

Polymerase chain reaction (PCR) is a molecular biology technology that can amplify a specific target DNA sequence in vitro. The PCR reaction requires repeated cycles at different temperatures to perform denaturation and annealing/extension of DNAs for the number of target DNA sequences to increase exponentially. Typically, the PCR reaction will execute 30–40 cycles [1,2]. Currently, the benchtop rapid PCR (e.g., Roche LightCycler 480 and Thermo Fisher Scientific Applied Biosystems 7500) have been able to complete 40 cycles within 30–40 min. Ultrafast PCR (<3 min, 40 cycles) requires a suitable biochemical environment and accurate and fast temperature-control system. In 2015, Wittwer et al., used experiments to confirm that the time limit of PCR reaction lies in the temperature-control system rather than the biochemical reaction kinetic [3,4,5]. Increasing the concentration of polymerase and primer in the reagent can provide the reaction kinetics required for the extreme PCR. The team used a water bath to verify the concept of extreme PCR. However, there is currently no commercial equipment on the market that can provide such a rapid temperature increase/decrease rate. The present study aims to achieve ultrafast PCR microfluidic chips suitable for practical application. The microfluidic techniques can reduce the number of reagents and samples used, speed up the reaction, and can be produced in large quantities to reduce costs [6,7,8]. Using this technology in ultrafast PCR, where the sample size is small and heat capacity is low, causes rapid temperature increase/decrease. Additionally, the sample can also have extremely high heat transfer efficiency in virtue of its high surface-to-volume ratio in the microchannel. In summary, microfluidic techniques are highly suitable for ultrafast PCR.

To date, various microfluidic techniques have been applied for PCR. Among these techniques, some may be used as heating and cooling platforms for ultrafast PCR, and some are unlikely to achieve ultrafast PCR in principle. Two design methods have been reported for microfluidic PCR: one is to make the sample position fixed, and the temperature changed in a single heating zone; the other is to move the sample between multiple heating zones, and the temperature of each heating zone is fixed. The microfluidic PCR with a fixed sample position must be equipped with a quick rising and falling temperature-control platform before it can be used in ultrafast PCR. It is almost impossible to meet the requirements of ultrafast PCR using block heaters to achieve fast temperature increase or decrease, as the heat capacity of the entire block is too high. Using infrared to heat a small-volume sample can achieve rapid temperature increase/decrease [9,10] because the aqueous-phase sample can easily absorb infrared wavelengths and heat up. Ouyang et al. used infrared rays to heat the fluid in the chip, and the heating and cooling rates were approximately 10 °C/s and 12 °C/s [11], respectively, which may meet the requirements of ultrafast PCR. However, light intensity and light source alignment must be paid attention to in applications of infrared heating methods. Additionally, a technology that uses an LED light source to excite the gold film in order to generate surface plasmon resonance to provide fluid with rapid temperature increase/decrease has been proposed [12]. This technology can provide temperature control with heating and cooling rates of approximately 12.8 °C/s and 6.6 °C/s, respectively, thereby completing a PCR thermal cycle in 10 s. This method has great potential for the temperature control of the ultrafast PCR platform. However, if fluorescent signals, such as real-time PCR, are used to detect target DNA sequences, avoiding the interference of the heating light source is necessary. In addition, two fluids with different temperatures are quickly injected into the independent cavity of the microfluidic chips to provide temperature control with the heating and cooling rates of approximately 25 °C/s and 18 °C/s [13,14], respectively. Nevertheless, this technology requires water baths of two temperatures, and instrument miniaturization is challenging.

Another rapid temperature-control strategy for microfluidic PCR is to move the fluid to circulate in multiple heating zones with a fixed temperature. As the temperature of the heating zone is fixed, the temperature-control system is relatively simple. For ultrafast PCR, this method should evenly conduct the heat source into the fluid effectively and move the fluid quickly. The implementation methods can be roughly divided into three categories. The first is called convective PCR, in which the heating source is placed under the PCR sample, and fluid heat conduction and convection are used to create the temperature gradient and fluid cycle, respectively [15,16]. This is the simplest PCR method in terms of hardware setup. The temperature required for denaturation is provided at the lowest end of the glass capillary, and the temperature will decrease from bottom to top to form a temperature gradient, and then reach the temperature required for annealing/extension. Due to convection, the fluid will be thermally circulated in the temperature gradient area in the capillary to achieve PCR, while its PCR efficiency is low. Additionally, the actual number of thermal cycles remains unknown, and the PCR speed cannot be too fast due to the limited speed of fluid convection. Another fluid control strategy is called oscillatory-flow PCR, or reciprocal-flow PCR, which allows fluid to move repeatedly in two to three temperature zones. The fluid can be controlled by injection pump or air pressure [17,18,19]. The advantage of this method is to reduce chip size, but it takes time for the sample to transfer back and forth between two or three temperatures. This fluid transfer time may be longer than the biochemical reaction time performed by ultrafast PCR. Additionally, some teams use the EWOD method [20] or magnetic fluid method [21] to drive the sample. In the EWOD method, the speed cannot be too fast, as the electrostatic force must overcome the friction force between the fluid and chip surface. Although the control method of the magnetic fluid is simple, the magnetic particles in the fluid cannot drive the movement of the entire fluid once the moving speed of the external magnet is too fast. Hence, these two fluid drive methods are unlikely to be used in ultrafast PCR.

The main concept of flow-through PCR is to sequentially heat and cool the samples between different temperatures in a single serpentine channel [22]. The advantage of flow-through PCR is that the chip design is simple, and so is the temperature-control system. Several flow-through PCR devices have been proposed in the past two decades [23,24,25]. However, few of them were focused on providing fast thermal cycles to achieve ultrafast DNA amplification, especially the chip substrate material that can provide fast heat transfer. In this study, we investigated the feasibility of a flow-through microchannel chip for ultrafast PCR application (<3 min, 40 cycles). A thin glass substrate was used to provide a suitable thermal conductor to transfer the heat source beneath the chip in a short time to the PCR sample. In addition, the design of the microchannel length and time required for denaturation and annealing/extension as well as platform detection limit were investigated.

## 2. Materials and Method

### 2.1. Chip Design and Fabrication

**Figure 1:**
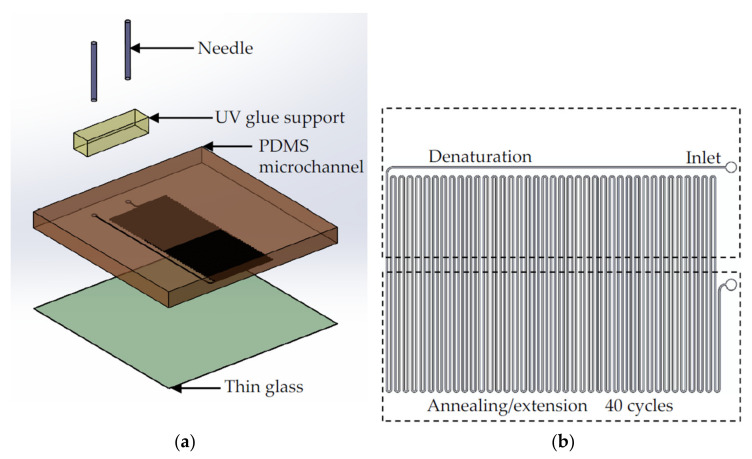
(**a**) Illustration of the flow-through ultrafast PCR chip. The chip is composed of PDMS and thin glass plate of 200 μm thickness, and a needle is inserted at the inlet and outlet of the sample. (**b**) The chip is designed with 40 thermal cycles and placed on the block heater of high-temperature zone (for denaturation) and low-temperature zone (for annealing/extension); the channel width is 200 μm, and the influence on ultrafast PCR is observed by changing the length of the channel.

The ultrafast flow-through PCR chip consisted of a polydimethylsiloxane (PDMS) layer and an ultrathin glass layer, as shown in Figure 1a. The microchannel was on the down side of the PDMS layer, and bonded to the 200 μm thick glass (AT35EX, Ruilong, Miaoli, Taiwan). The 25G needle was connected to the fluid inlet and outlet of the PDMS layer, and UV glue (5 Second Fix, Ontel, Fairfield, NJ, USA) was used to bond the needle to the PDMS. The microchannel structure was defined by the master mold made on four-inch silicon wafer (100 mm, Summit-Tech, Taiwan). The fabrication of the master mold used a laminator (AL-320, Allpass, Taiwan) to attach photosensitive dry films (56 μm HM-4056, Showa Denko Materials, Tokyo, Japan) to silicon wafers, and then the photomask was pressed onto the dry film photoresist. The operation procedure of the dry film photoresist followed the vendor’s instructions. The dry film photoresist was placed in Na_2_CO_3_ (1%, Maxwave Co., Taipei City, Taiwan) for development. After the development was complete, deionized water was used to remove the remaining developer. It was dried with nitrogen, placed on a hotplate at 120 °C, and baked for 6 min to complete the master mold. The PDMS structure layer was made using the standard soft lithography process, and PDMS and glass were bonded via an oxygen plasma (PDC-001, Harrick Plasma, Ithaca, NY, USA) with a power of 30 W and oxygen pressure of 0.05 Torr. Figure 1b shows the design of the flow-through ultrafast PCR chip. The channel width and interchannel distance were 200 and 400 μm, respectively; 40 thermal cycles were conducted, and denaturation and annealing/extension were achieved in high- and low-temperature zones, respectively. The channel lengths of 6, 4, and 2.4 cm per cycle with a height of 56 μm were used to investigate the effects on ultrafast PCR.

### 2.2. Experimental Setup and Design

**Figure 2:**
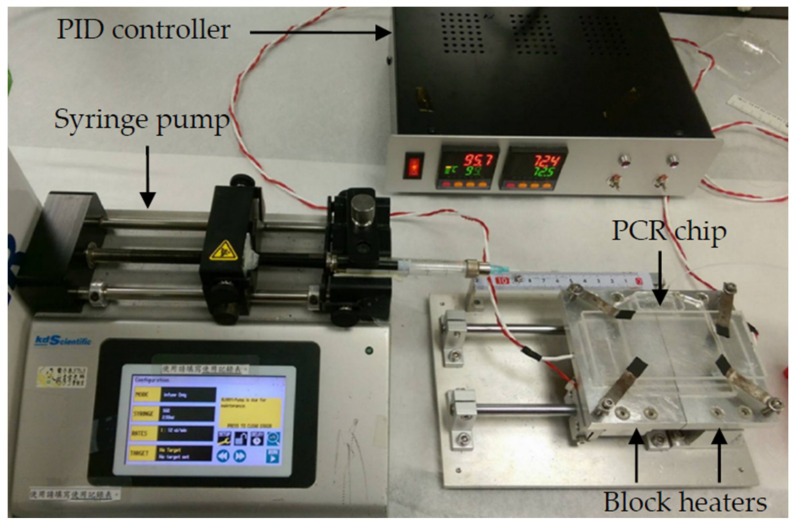
Experimental setup of ultrafast PCR platform. The block heaters at the bottom of the chip use a polyimide film heater for heating and a PT100 temperature sensor for measuring the temperature. The temperature is controlled via PID feedback, and the sample is injected into the chip via the syringe pump.

The experimental setup of the ultrafast PCR platform consisted of the temperature-control system, fluid-control system, and microfluidic chips, as shown in Figure 2. The prepared microfluidic chips were placed on two individual block heaters to allow the denaturation and annealing/extension of the DNA. To speed up the PCR, the temperatures required of the reagent for annealing and extension were adjusted to be close. Hence, only two temperatures were needed for PCR. The block heater was made by attaching a polyimide film heater (KP100100R125, MIYO Technology Co., New Taipei City, Taiwan) to the bottom of the aluminum block, embedding the temperature sensor PT100 in the aluminum block, and accurately controlling the temperature through PID (FY400-20100B, Taiwan Instrument & Control Co., New Taipei City, Taiwan), with an air gap of 1 mm between the two heating aluminum blocks. A syringe pump (Legato 180, KD Scientific Inc., Hilliston, MA, USA) was used as a driving force of fluid. An infrared thermal imaging camera (A40, Teledyne FLIR, Waterloo, ON, Canada) was used to measure the temperature distribution on the block heater and chip. The procedure of the ultrafast PCR was to first fill the entire channel with perfluoropolyether oil (Fomblin Y04, SOLVAY, Brussels, Belgium), and then fill the syringe with 1 mL of perfluoropolyether oil, and finally to extract 10 μL of the PCR sample. The sample was injected into the chip performing the PCR, which covered the front and back ends of the sample with fluorinated oil, increasing the pressure in the microchannel to suppress the bubbles generated when the sample passed through the high-temperature zone [26,27]. After the execution of 40 thermal cycles, the sample was collected from the outlet and gel electrophoresis was performed to confirm the product. The channel had designs of three lengths to find the suitable design parameters. Additionally, the optimization of denaturation and annealing/extension temperature and the time ratio of fluid in the high-temperature zone to the low-temperature zone were studied.

### 2.3. Sample Preparation and Gel Electrophoresis

The template DNA for the establishment of the ultrafast PCR platform was genomic DNA from the leukemia cell line K562. The target amplicon was a 151-base-pair fragment in the actin gene, which is mostly used as a housekeeping gene in genetic testing. The reaction mixture (10 μL) contained 50 mM Tris–HCl (pH 8.5), 3 mM MgCl_2_, 500 μg/mL BSA, 3 μM forward primer (5′-ATG GTG GGA ATG GGT CAG AAG-3′), 3 μM reverse primer (5′-GCA GCT CAT TGT AGA AGG-3′), 10 ng genomic DNA template, 7.5 U Klen *Taq* DNA polymerase (PT-GL-KTAQ, Protech Technology Enterprise Co., Taiwan), and 0.01% (*w*/*v*) bromophenol blue. These components were mixed and injected into the chip to perform ultrafast PCR, as described in Section 2.2. After PCR, the products were verified via gel electrophoresis. Specifically, the agarose was dissolved in Tris–acetate–EDTA buffer to prepare 2% agarose gel, which was poured into a colloid preparation tank and solidified at room temperature. The PCR product was mixed with 1 µL of loading dye and loaded into the agarose gel. DNA electrophoresis was performed at 100 V for approximately 30 min. The copy number of the actin gene was calculated as follows. One genome of K562 cell line DNA is approximately 3 pg, in which there are around 20 copies of actin genes. Therefore, 10 ng genomic DNA has approximately 67,000 copies of actin genes (10 ng/3 pg × 20 copies = 66,667 copies).

## 3. Results and Discussion

### 3.1. Temperature and Fluid Control

An infrared thermal imager was used to measure the uniformity of the surface temperature of the thin glass, which was coated with a high emissivity polymer on the surface and placed on two independent temperature block heaters. The high- and low-temperature zones were set to 99 °C and 72.5 °C, respectively, and the two block heaters were separated by 1 mm. Section 3.2 will describe the reason for using these temperature settings. The results are shown in Figure S1. The temperatures of the thin glass on the two block heaters were highly uniform. The actual temperature in the microchannel is difficult to measure, although it is important information for the study. Hence, the surface temperature of the glass substrate was measured to estimate the temperature the sample would contact. The measured temperature was calibrated using a reference thermal couple attached to the glass substrate. We attempted to measure the actual temperature in the microchannel through the use of fluorescent dye as a thermal indicator. Note that two-temperature PCR is becoming popular as it can simplify the design of devices and provide comparable efficiency to the traditional three-temperature PCR [28]. The two temperatures are usually 95 °C for denaturation and 60–65 °C for annealing and extension. To apply two-temperature PCR, longer primers are required to have annealing temperature higher than 60 °C. In addition, the DNA polymerase needs to work at suboptimal temperatures. Although the extension temperature of the two-temperature PCR is lower than typical use, i.e., 72 °C, the performance of the DNA polymerase was acceptable in our experiments.

**Figure 3:**
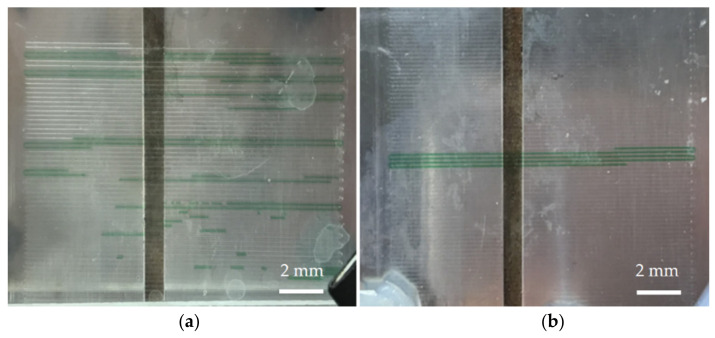
Fill the channel with fluorinated oil and encapsulate the sample in the oil phase; increase the pressure of the fluid in the microchannel to suppress the generation of bubbles. (**a**) When the sample is not encapsulated with oil, the sample passes through the high-temperature zone and generates bubbles, causing in the destabilization of flow. (**b**) When the sample is encapsulated in the oil phase, the bubbles are suppressed, causing the stabilization of flow.

In terms of fluid control, if the fluid is pushed by the air in the syringe when the chip is not heated, the flow rate of the fluid is not stable due to the compressibility of the air. If the oil-phase fluid is used to push the aqueous-phase samples, the instability of the flow rate can be improved. However, most DNA samples have a denaturation temperature above 90 °C, which is close to the boiling point of water. We used oil-phase fluid to push dyed ink in the microchannel and found that when the aqueous-phase fluid passes through the high-temperature zone, bubbles are generated and it expands, causing the destabilization of flow, as shown in Figure 3a. The open outlet of the channel can deduce that the pressure in the channel is not enough to resist the vapor pressure generated by the aqueous-phase fluid being exposed to the high temperature. Therefore, before the aqueous-phase sample entered the channel, the channel was filled with perfluoropolyether oil and then the perfluoropolyether oil in the syringe was used to push the aqueous PCR sample into the channel, and the oil phase encapsulating the aqueous PCR sample was formed. In addition to using the oil to fill the small holes in the channel wall, the friction between the perfluoropolyether oil and channel wall increased the pressure in the entire channel and inhibited the generation of bubbles. The larger the contact area, the higher the pressure generated in the microchannel. Moreover, the boiling point of oil is higher than that of the aqueous-phase sample. We used dye ink as the aqueous phase reagent and the oil phase encapsulating the aqueous phase to push the sample, and the result is as shown in Figure 3b: the use of the sample encapsulation in oil can greatly reduce air bubbles. Thus, the aqueous-phase fluid can be stably pushed in the heated channel.

### 3.2. Effects of Denaturation and Annealing/Extension Temperature on Ultrafast PCR

**Figure 4:**
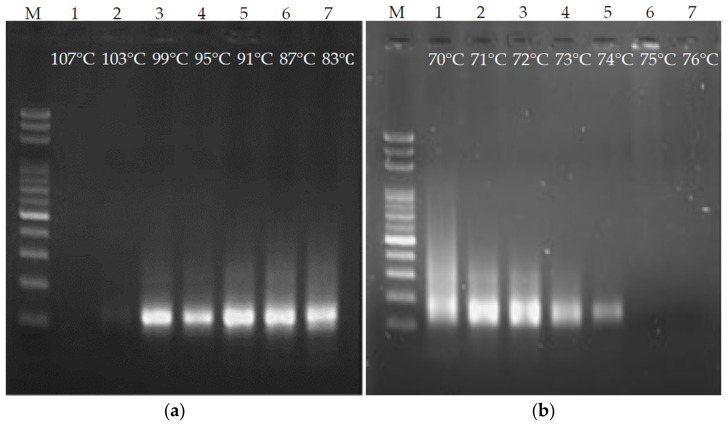
Temperature optimization of denaturation and annealing/extension of ultrafast PCR. The suitable temperature for (**a**) denaturation is between 99 °C and 83 °C and (**b**) annealing/extension is approximately 73 °C.

Temperature is a crucial factor in order for PCR to be carried out. Inappropriate temperature can hinder PCR or lead to the generation of nonspecific products. In particular, fluids stay at each temperature for a very short time on the ultrafast PCR platform. Hence, denaturation and annealing/extension temperatures were investigated. First, the annealing/extension temperature was fixed at 72 °C, the denaturation temperature was used as the variable, and ultrafast PCR was performed every 4 °C from 107 °C to 83 °C. The time for fluid to pass through the denaturation and annealing/extension areas was set to 3 s for each, and gel electrophoresis was used for the PCR product evaluation. As shown in Figure 4a, when the denaturation temperature was 103 °C, the product band on the gel image was very weak. There was no product at 107 °C. It is inferred that if the denaturation temperature was set over-high, the PCR enzyme reaction would be inhibited. The designated products were obtained at 99–83 °C. However, the lower the temperature, the more smeared products (nonspecific products) on the gel image. Hence, the denaturation temperature was set to 99 °C. In the terms of the annealing/extension temperature, ultrafast PCR was performed every 1 °C from 76 °C to 70 °C, with the denaturation temperature fixed at 99 °C and the time for the fluid to pass through the denaturation and annealing/extension areas set to 3 s (Figure 4b). When the annealing/extension temperature exceeded 75 °C, no PCR product was produced. It is speculated that the primer cannot attach to the DNA due to the high temperature. When the temperature setting was lower than 72 °C, the smear phenomenon became more serious, so the annealing/extension temperature was set to 72.5 °C.

### 3.3. Effects of Flow Rate and Microchannel Length on Ultrafast PCR

**Figure 5:**
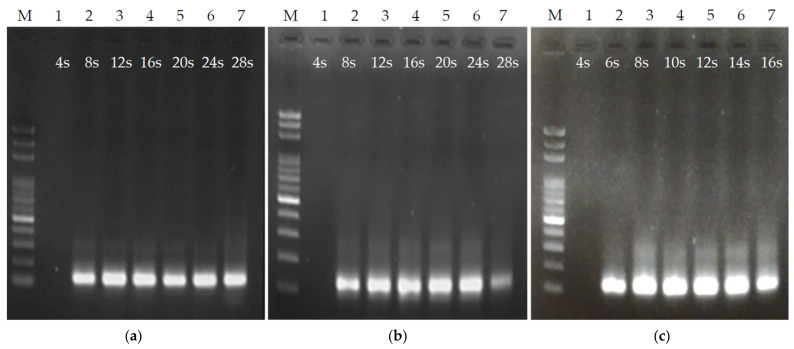
Ultrafast PCR tests under different channel lengths and flow rates. (**a**,**b**) PCR results with a 56 μm channel height and a channel length of 6 and 4 cm/cycle, with each thermal cycle lasting 4–28 s. (**c**) PCR results with a 56 μm channel height and a channel length of 2.4 cm/cycle, with each thermal cycle lasting 4–16 s.

The channel height was fixed at 56 μm and microchannels with lengths of 6, 4, and 2.4 cm for each thermal cycle were made. Note that we tried to use a microchannel with a height of 27 μm and found that the flow resist was too large, so the fluid could not be injected smoothly via the syringe pump. The fluid passed through the denaturation and annealing/extension areas for half the time. The flow rate of the syringe pump was tuned to adjust the time of the PCR sample flow through each thermal cycle from 4 to 28 s. Gel electrophoresis was performed to verify the ultrafast PCR at every 4 s interval. The results of the ultrafast PCR for each flow rate under different channel lengths were observed. Figure 5a shows the gel image results of PCR with a length of 6 cm in each thermal cycle under different sample flow rates. It can be seen that no product appeared in the PCR with each thermal cycle of 4 s, and there were products with each thermal cycle of 8, 12, 16, 20, 24, and 28 s. The chip with a length of 4 cm in each thermal cycle had similar results to the 6 cm chip, as shown in Figure 5b. However, in the 4 cm chip, the gel image result of the PCR with each thermal cycle of 28 s had lighter and higher band positions than other conditions. It was observed that under this operating condition, the samples generated a few bubbles when flowing through the high-temperature zone, which made the flow rate unstable. It is speculated that the flow rate of the fluid was too slow under this operating condition, so that the pressure in the microchannel was too small to suppress the vapor pressure generated by the fluid at high temperatures [26,27]. The modified Hagen–Poiseuille equation that is suitable for the rectangular cross-section of the microchannel was used:

(1) $$\Delta p\approx Q\frac{12\eta L}{h^{3}w(1-0.63h/w)}$$

where ∆*p* is the pressure difference formed by the fluid in the channel, *Q* is the flow rate, *h* is the microchannel height, *w* is the microchannel width, *L* is the length of the fluid in the channel, and *η* is the fluid viscosity coefficient. The faster the flow rate *Q*, the more the fluid pressure ∆*p* increases in the channel. The sample flow rate is set to 1.54 μL/min in 4 cm/cycle chip and the sample residence time of 28 s/cycle, which is slower than the flow rate of 2.31 μL/min in 6 cm/cycle chip and the same sample residence time. Figure 5c is the result of the chip PCR gel image with a length of 2.4 cm in each thermal cycle. As there was no PCR product under the operating conditions of 4 s in each thermal cycle, the designated products can be obtained under the remaining 6 s to 16 s. Under this channel length, to obtain the same sample residence time as the above two channel length designs, the required flow rate is slower. When each thermal cycle exceeds 16 s (with a flow rate of 1.62 μL/min), a large number of bubbles appeared in the fluid in the high-temperature zone, which resulted in an unstable flow rate. This result is similar to the situation described above with the sample residence time of 28 s with a length of 4 cm in each thermal cycle. Therefore, the PCR was only tested within 16 s of each thermal cycle. From the above results, the time for fluid to pass through the heating zone can be controlled by channel length and fluid flow rate. An important consideration in channel length design is that it should not be overly short, as otherwise the flow rate of the fluid will be too slow, causing overly low pressure in the channel and the generation of bubbles in the high-temperature zone, which subsequently causes an unstable flow rate.

### 3.4. Influence of Time Ratio of Sample Flow-Through Denaturation and Annealing/Extension on Ultrafast PCR

**Figure 6:**
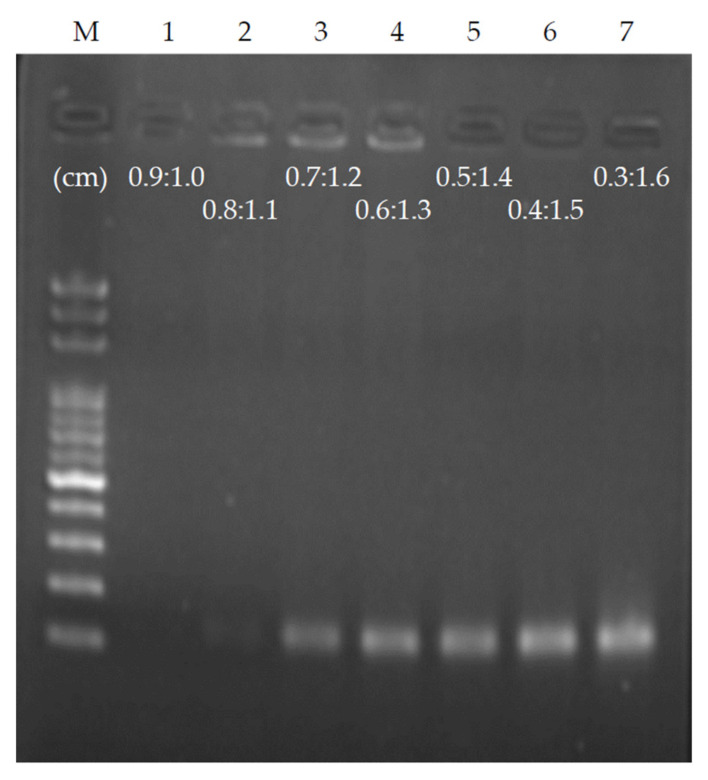
PCR result of changing the length (time) ratio of denaturation and annealing/extension and fixing the flow rate of each 4 s thermal cycle. When the ratio of denaturation and annealing/extension is adjusted to 0.7:1.2, the products appear. The sample residence time of annealing/extension must be sufficient to allow the reaction to complete.

As the PCR requires a short keeping time during denaturation, the double-stranded DNA will denature as long as the sample temperature is higher than the melting point of the DNA, and the time required for primer annealing on the template is also less than 1 s [4]. The entire PCR takes the longest time in extension. To optimize the time for ultrafast PCR samples to flow through the denaturation and annealing/extension areas, we used a chip with a height of 56 μm and a length of 4 cm in each thermal cycle, and fixed the flow rate so that the time of each thermal cycle was 4 s. The ratio of denaturation to annealing/extension was set to 0.9:1.0 as the benchmark, denaturation was sequentially reduced by 0.1, and annealing/extension was sequentially increased by 0.1 until the ratio was 0.3:1.6. A total of seven conditions were observed, shortening the sample residence time of denaturation, extending the sample residence time of annealing/extension, and performing ultrafast PCR for each ratio (Figure 6). Under the condition of a ratio of 0.9:1.0 and each thermal cycle of 4 s, PCR products could not be obtained. As the sample residence time of annealing/extension increased, PCR products were generated at the ratio of 0.7:1.2. Once the ratio reached 0.3:1.6, maximum PCR products were obtained. It can be estimated that the minimum time required for the denaturation on the chip is approximately 0.63 s; the minimum time required for annealing/extension is approximately 2.53 s. Hence, it is possible to reduce the PCR time to 3.16 s per thermal cycle using the proposed chip. As the length of target DNA sequences is 151 base-pairs (bps), it can be estimated that the fastest DNA extension rate in this chip can reach approximately 60 bps/s, which is consistent with other PCR studies using the same Klen Taq (50–100 bps/s) [3,4].

The heat transfer time required for the reagent under this platform is extremely short, which is very suitable for the development of an ultrafast PCR chip. Comparing our experimental results to the other studies performing ultrafast PCR using continuous-flow-based microfluidic devices (Table 1), our platform may provide a faster PCR time. This can be attributed to the higher thermal conductivity of the material of the chip substrate. The heat source is provided under the microfluidic chip, so the heat transfer rate from the heat source to the reaction sample passing through the chip substrate is a crucial consideration for ultrafast PCR. The thin glass plate used in this study has approximately 1 W/m-K thermal conductivity, which is higher than other setups that use polyimide, FR4 PCB, or polycarbonate substrate. In terms of heater spacing, the determination of the spacing between the two heaters is a tradeoff between some factors. For example, larger is better for cooling from the high-temperature zone to the low-temperature zone because the cooling duration needs more nature cooling time. In contrast, a shorter distance is better for the temperature-raising process because when the fluid flows from the low-temperature zone to the high-temperature zone, the sample temperature first decreases due to nature cooling at heater spacing, which should be avoided. In addition, the lateral heat conduction should also be considered for the spacing between the heaters, which may smear the temperature zones. In our observation, for ultrafast PCR purposes, the actual temperature of the sample in the microchannel may not be necessarily be equal to the set of the heater. It is a dynamic equilibrium of temperature in the microchannel. Once the equilibrium temperature is suitable for reaction, PCR takes place. The equilibrium temperature in the microchannel can be tuned by the two heaters. As a result, the spacing between the high and low-temperature zones in our setup is quite narrow for the sample passing quickly through the transition zone of the two temperatures. Furthermore, we attempted to measure the actual temperature in the microchannel using fluorescent dye for a better understanding of the ultrafast feature.

### 3.5. Detection Limit of the Chip

**Figure 7:**
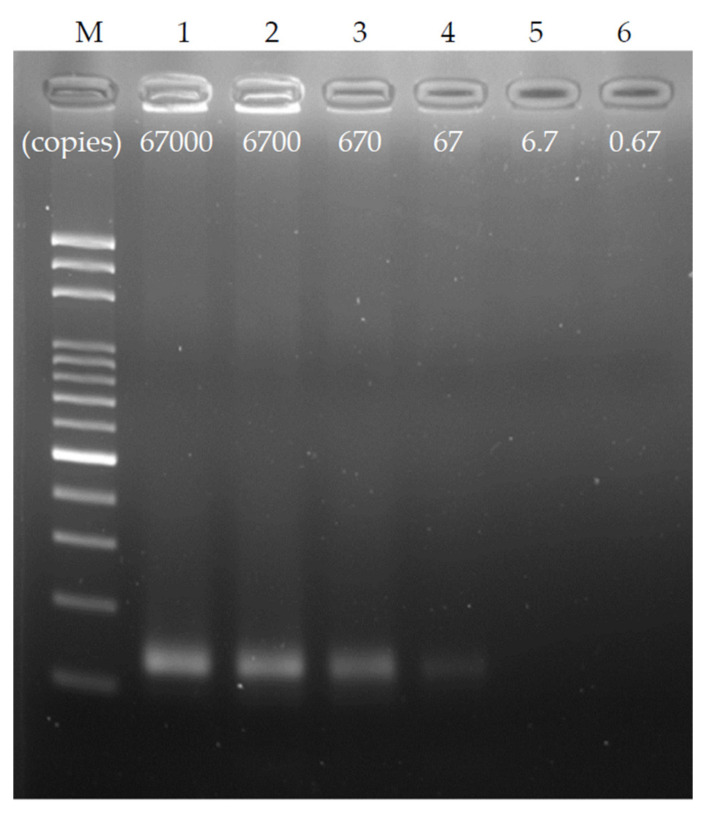
Ultrafast PCR detection limit; the original concentration of 67,000 copies of the sample was diluted with a 10-fold sequence and tested with a flow rate of 6 s/cycle. It was found that the detection limit under this PCR condition was approximately 67 copies, which was equivalent to 40 thermal cycles at 95 °C for 2 s and 72 °C for 4 s in the water tank via manual operation.

To explore the detection limit of the chip, a chip with a channel width of 200 μm, a height of 56 μm, and a length of 4 cm per thermal cycle was used, and the ratio of denaturation to annealing/extension was set to 0.3:1.6. To ensure the stability of the test, a single thermal cycle time was set at 6 s, and the DNA sample was diluted by a 10-fold sequence from the original copy number of 67,000 copies (10 ng genomic DNA) to 10^–5^ times that, at 0.67 copies (50 fg). Figure 7 shows the dilution result of the ultrafast PCR sequence using gel electrophoresis. As observed, clear products were obtained in both 67,000 and 6700 copies. After 670 copies, the brightness of the product band decreased, and the weak brightness of the product band can be seen under 67 copies. Therefore, it is speculated that the detection limit of this chip under the above operating conditions can reach 67 copies or one aM. This result is equivalent to 40 thermal cycles at 95 °C for 2 s and 72 °C for 4 s in the water tank via manual operation, and it has high PCR efficiency.

## 4. Conclusions

Since the time limitation of PCR is not in the biochemical reaction kinetics, a stable platform that can provide quick temperature response in reagent is the key to the commercialization of ultrafast PCR. This study used a flow-through PCR channel design to achieve an ultrafast PCR and discussed the length of microchannel and the temperatures of denaturation and annealing/extension, as well as the time ratio of denaturation to annealing/extension. It was found that the design of the channel length needs to cooperate with the sample residence time, flow rate setting, and pressure in the channel. A channel with an overly short length will require low drive-flow rates. As a result, the pressure in the channel is not enough to suppress the generation of bubbles and the fluid becomes unstable. In terms of DNA denaturation by the chip, the chip only needs 0.63 s to achieve complete denaturation of the DNA, and the residence time is provided to the sample for heat conduction. The minimum time required for annealing/extension is approximately 2.53 s. Hence, the optimal time ratio of denaturation and annealing/extension via the chip is approximately 1:4. The operation window of the annealing/extension temperature and the sample residence time is relatively small compared with denaturation. The annealing/extension temperature must be optimized for each reagent. The optimal temperature required for different primer designs and different templates will be different, and the sample residence time depends on the length of the amplicon and the polymerase enzyme used. The use of the chip with Klen Taq enzyme can provide an extension speed of approximately 60 bps/s, which is equivalent to the theoretical value of 50–100 bps/s for the extension of the polymerase enzyme. The chip provides very fast thermal exchange speed. In summary, the proposed chip can complete 40 thermal cycles of PCR in 160 s, and the detection limit can reach 67 copies. The chip is made of PDMS and ultrathin glass, is easy to manufacture, and is simple to design. It is promising for a commercial platform of ultrafast PCR.

## Figures and Tables

**Table 1:** Comparison of the experimental result to similar studies performing ultrafast PCR using continuous-flow-based microfluidic devices.

Minima Time Required Per Cycle	Sample Volume	Amplicon Size (Base Pair)	Polymerase	Chip Material	Thermal Conductivity of the Substrate Material (W/m-K)	Ref.
10 s (300 s/30 cycles)	25 μL	90	KAPA2G Fast DNA polymerase	All polyimide	0.12 (polyimide)	[29]
4 s (120 s/30 cycles)	30 μL	157	KAPA2G Fast DNA polymerase	PCB substrate with photoresist	0.25 (FR4 PCB)	[30]
16 s (480 s/30 cycles)	NA	311	SpeedSTAR HS DNA polymerase	All polycarbonate	0.2 (polycarbonate)	[24]
4 s (160 s/40 cycles)	10 μL	151	Klen Taq DNA polymerase	PDMS with glass substrate	1 (glass)	This work

## Supplementary Materials

The following are available online at https://www.mdpi.com/article/10.3390/bios12050303/s1, Figure S1: Infrared thermal imaging camera was used to measure the temperature distribution on the thin glass. The thin glass was placed on a heating platform with two temperatures. The left and right sides were the high- and low-temperature zones, respectively, and the dotted box was the range of the glass. Six positions were randomly taken for temperature measurement at each temperature zone. With a standard deviation of 0.15, the average temperatures were 99.1° C and 72.0 °C at the high- and low-temperature zones, respectively. The temperature of the glass on the two block heaters is uniform.
