# Supplementary material for: Ultrafast DNA Amplification Using Microchannel Flow-Through PCR Device
Source: Biosensors (Basel). 2022 May 6;12(5):303. doi: 10.3390/bios12050303 (PMC9138433; doi:10.3390/bios12050303)
Supplement: Supplementary file 1 [file biosensors-12-00303-s001.zip › biosensors-1686463-supplementary.pdf]

biosensors

MDPI

Ultrafast DNA Amplification Using Microchannel Flow-Through PCR Device

Yen-Heng Lin 1,2,* , Xiang-Jun Liao 1, Wei Chang 3 and Chiuan-Chian Chiou 3,4,5,*

[1] Graduate Institute of Biomedical Engineering, Chang Gung University, Taoyuan 333, Taiwan; hanerbill@gmail.com
[2] Department of Laboratory Medicine, Chang Gung Memorial Hospital, Linkou, Taoyuan 333, Taiwan
[3] Master and PhD Program in Biotechnology Industry, College of Medicine, Chang Gung University, Taoyuan 333, Taiwan; deamit114@gmail.com
[4] Department of Medical Biotechnology and Laboratory Science, College of Medicine, Chang Gung University, Taoyuan 333, Taiwan
[5] Department of Thoracic Medicine, Chang Gung Memorial Hospital, Linkou, Taoyuan 333, Taiwan

* Correspondence: yenheng@mail.cgu.edu.tw (Y.H.L.); ccchiou@mail.cgu.edu.tw (C-.C.C.)

Supplementary Information

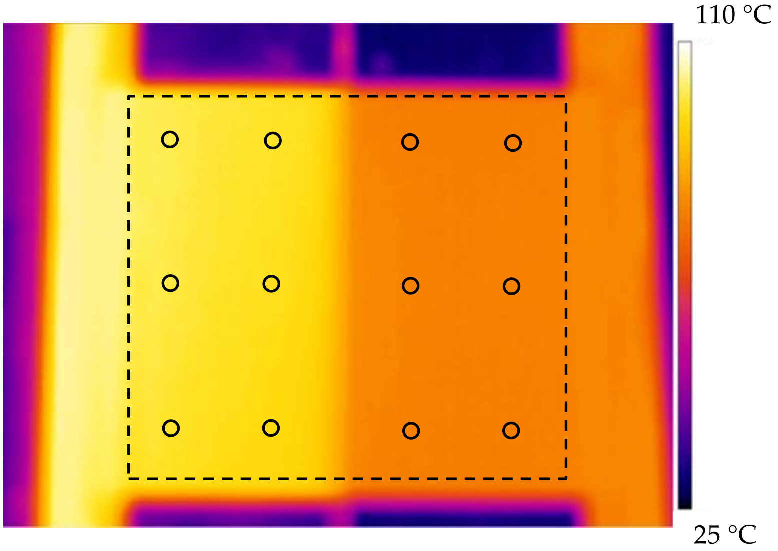

Figure S1. Infrared thermal imaging camera was used to measure the temperature distribution on the thin glass. The thin glass was placed on a heating platform with two temperatures. The left and right sides were the high- and low-temperature zones, respectively, and the dotted box was the range of the glass. Six positions were randomly taken for temperature measurement at each temperature zone. The average temperatures were 99.1°C with a standard deviation of 0.15 at the high-temperature zone and 72.0°C with a standard deviation of 0.15 at the low-temperature zone. The temperature of the glass on the two block heaters is uniform.
